# The link between animal personality and habitat selection in males of the Neotropical poison frog *Allobates femoralis*

**DOI:** 10.1163/1568539X-bja10202

**Published:** 2023-01-18

**Authors:** Lauriane Bégué, Mélissa Peignier, Eva Ringler

**Affiliations:** a Division of Behavioural Ecology, Institute of Ecology and Evolution, University of Bern, Wohlenstrasse 50a, CH-3032 Hinterkappelen, Switzerland; b Department of Biology and Ecology, University of Montpellier, 2 place Pierre Viala, 34060 Montpellier, France

**Keywords:** boldness, captive population, exploration, habitat choice, habitat complexity, novel environment test

## Abstract

Environmental variation plays a key role in the evolution and maintenance of animal personality. Individuals with different personality types might exhibit different habitat preferences. Alternatively, variation in individual behaviour across space could arise as a plastic adaptation to distinct habitats. Our study aims to investigate if habitat choice is influenced by an individual’s personality. We assessed individual levels of activity, boldness, and exploration in male poison frogs, and performed a habitat choice test under controlled laboratory conditions. Individuals were consistent in their behaviours, but all tested frogs chose the complex over the simple habitat. Individuals that were characterized as bold and very explorative also showed more movements between the two different habitats in the choice test. These results indicate that personality measured in a highly standardized artificial setup, such as a novel environment test, indeed can reflect boldness and exploration related behaviours measured in a more naturalistic setup.

## 1 Introduction

Empirical evidence for the existence of consistent behavioural differences between individual animals across time and contexts, also termed ‘animal personality’, has been found in various different taxa (reviewed in Réale et al., 2007; Gosling, 2008). Yet, we do not fully understand how animal personality arises and is maintained in animal populations (DiRienzo & Montiglio, 2015). The selective pressures induced by environmental heterogeneity have been proposed as potential mechanisms both generating and maintaining individual differences in behaviour within a population (Dingemanse et al., 2004). Situations where personality traits are non-randomly distributed across the natural and social environment are referred to as ‘phenotype by environment correlation’ (Conover & Schultz, 1995; Dingemanse & Araya-Ajoy, 2015). Unfortunately, to date very few studies have investigated whether habitat selection could be driven by animal personality, or whether behaviours are adjusted in response to the environmental conditions an individual is, or has been, exposed to (i.e., behavioural plasticity).

A study in dunnocks (*Prunella modularis*) has shown that bold individuals settled in areas with high human disturbance, and that individuals also became bolder with age (Holtmann et al., 2017). The effect of behavioural plasticity was weak compared to the effect of personality, which provides first evidence that personality was the most important factor determining the individuals’ distribution across the habitat. More empirical research is needed to better understand the processes that influence how animal personalities are distributed across the environment.

The existence of personality has been demonstrated in several species of amphibians (reviewed in Kelleher et al., 2018), and amphibians are typically distributed across heterogeneous environments making them excellent model species to investigate the distribution of personalities across the natural environment. A recent study in a wild population of the Neotropical poison frog *Allobates femoralis* showed that males exhibit consistent within- and between-individual variation in territorial aggression, boldness and exploration (Peignier et al., 2022). There was no link between males’ personality traits and properties of their natural environment known to affect sound transmission and visibility to females and predators (i.e. overall vegetation complexity in the area surrounding the male’s territory and also territory size). But males seemed to adjust their level of exploration and boldness in response to changes in their social environment, especially to the density of females nearby. During the breeding season, males need structures such as branches, logs and roots where they perch during advertisement calling (Ursprung et al., 2011; Ringler et al., 2012) and suitable leaves for clutch deposition (Ringler et al., 2013; Ringler et al., 2018). In the present study, we thus asked if males with different behavioural phenotypes exhibit contrasting habitat preferences in terms of fine scale vegetation structure during territory establishment.

We tested if habitat complexity drives habitat choice in *Allobates femoralis* males, and further checked if habitat preferences are linked to specific personality traits. We did not consider females in our study, because they only show site fidelity to resting sites (Fischer et al., 2020) from where they visit male territories for courtship and mating (Ringler et al., 2012). As space use in females is mainly driven by mating, we assume that the social environment (i.e. males in the surrounding) rather than complexity of the natural environment plays a role for habitat choice in female *A. femoralis*. We focused on 36 males from a captive population of *A. femoralis* to quantify within- and between-individual consistency in activity, boldness and exploration. We investigated habitat selection by presenting males with a two-choice test between a non-complex (i.e., with fewer hiding places, perches, water bodies and leaf litter) and a complex habitat. When faced with a novel environment, we expected males to either all choose the same habitat — which would reflect an ideal habitat for the species — or select a specific habitat according to their personality. In the latter case, we expected bolder individuals to occupy areas of low complexity (i.e. sparse vegetation and few ground structures) where they are easier to spot for females, while shyer individuals are expected to occupy areas of higher complexity with more places to hide.

## 2 Material and methods

### 2.1 Study species and experimental setup

The brilliant-thighed poison frog, *Allobates femoralis*, is a small diurnal leaf litter frog common throughout the Amazon basin and the Guiana Shield (Amézquita et al., 2009). During the reproductive season, males emit prominent advertisement calls from elevated structures on the forest floor (e.g., branches, logs, etc.) to announce territory possession to conspecifics and attract female mating partners into their territory (Hödl et al., 2004). Males generally occupy territories ranging from 64.62 to 417.63 m^2^ in size (Ringler et al., 2011). Females are not territorial and commute to males’ territories for courtship and mating (Ringler et al., 2012; Fischer et al., 2020). After tadpole hatching, males transport the larvae to water bodies located up to 200 m away from their territories (Ringler et al., 2013, 2018).

We conducted our study in spring 2021 under controlled laboratory conditions in the animal care facilities at the Ethological station of Hasli from the University of Bern. The studied population consists of wild caught frogs from French Guiana and captive bred frogs. Individuals are kept in breeding pairs in standard (60 × 40 × 40 cm) glass terraria furnished with a coconut shelter, a perch, a plant, a water bowl, and expanded clay pebbles covered with autoclaved oak leaves. The sides are covered with Xaxim (tree fern stems) mats in the lower half and cork in the upper half to prevent visual contact between terraria. Light, temperature and humidity are automatically controlled to mimic natural conditions in French Guiana.

All behavioural tests were performed on 36 adult males during their reproductively active period (November–June). Individuals were unambiguously identified via their unique ventral coloration patterns (Ringler et al., 2014). We photographed all males over millimetre paper and measured their body size by determining their snout to urostyle length (SUL) using the software ImageJ (Rasband, 1997–2021).

We assessed individual levels of activity, boldness and exploration (see below) for all focal males, by repeating each trial three times per individual (cf. Réale et al., 2007) resulting in a total of 108 tests per assessment. Boldness and exploration were tested within the same setup. To prevent habituation and/or fatigue during the experiments, no individual was tested twice on the same day, and we ran activity assessment and boldness/exploration assessment on separate days (cf. Uher & Asendorpf, 2008; Roche et al., 2016). We tested individuals in a semi-random fashion (e.g., either activity or boldness/exploration assessment first). On average two tests of the same assessment were separated by 7.5 days (range = 1–19).

### 2.2 Assessment of activity levels

Similar to the behaviour of *A. femoralis* individuals in the wild, males in our laboratory population are usually more active during the afternoon and particularly after rainfall (Aichinger, 1991; Ringler et al., 2013). Therefore, we ran activity trials immediately after the daily activation of the raining system in the afternoon, from 1600 to 1900. To this end, we selected six breeding pairs per day, where we removed the respective females from the tanks and placed a wide-angle video camera (Hero Black 5 and 8, GoPro, San Mateo, CA, USA) on top of each terrarium. The first 45 min of recording were not considered in the analysis to allow individuals to resume normal behaviour after the experimenter had exited the room. Temperature in the room during those trials was constant (28,3 ± 0.4°C).

We analyzed the subsequent 60 min of video recordings using the coding software BORIS (Friard et al., 2016). We coded activity as: (i) number of jumps and (ii) call duration (in s). The setup of the video cameras allowed us to monitor the entire space inside male terraria. It occasionally occurred that visibility of males was slightly obstructed by the vegetation, but in general male movements were unambiguously visible. Male calls were recorded at all times. In instances where males were neither visible nor audible for the entirety of the recording (e.g., hiding under a shelter, *N* = 27/108 observations), we coded them with a value of zero for both number of jumps and call duration.

### 2.3 Assessment of boldness and exploration levels

**Figure 1:**
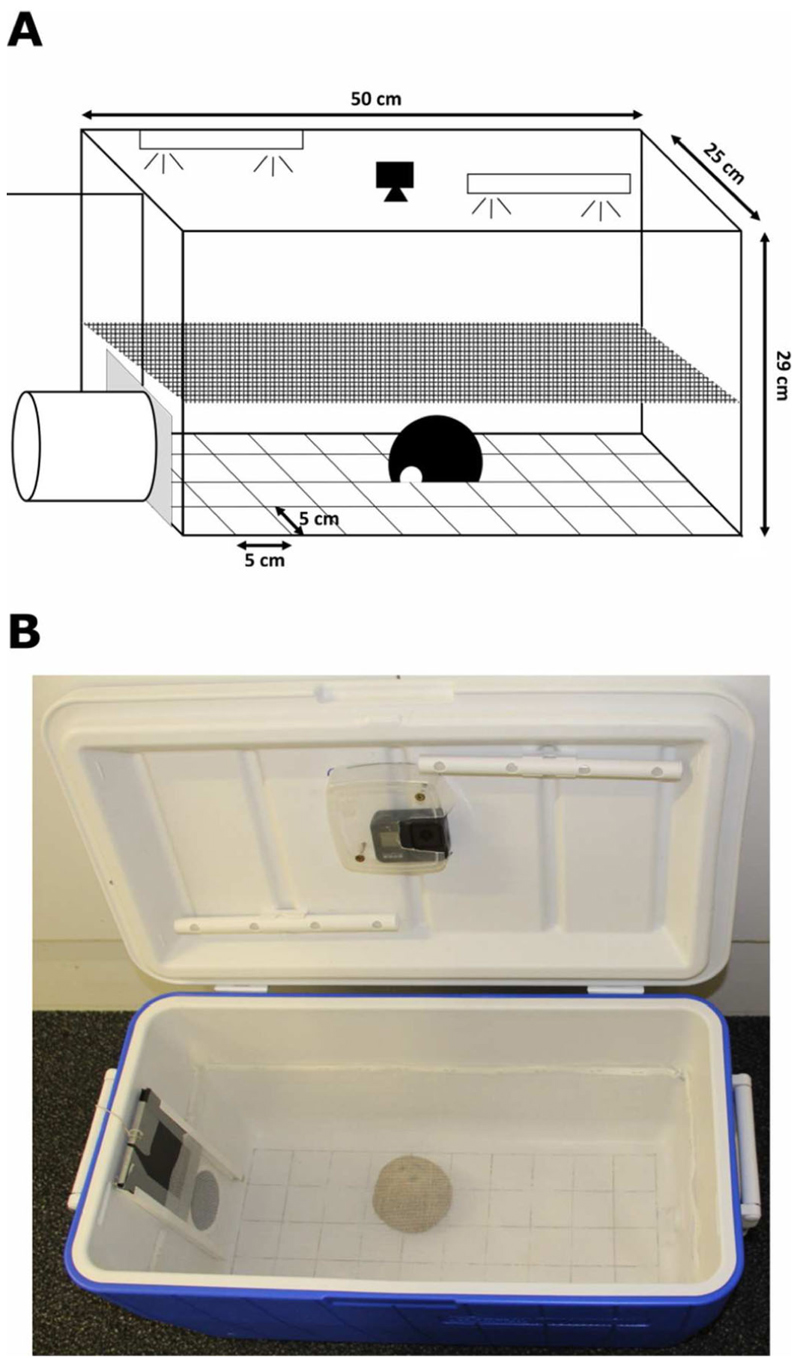
Scheme (A) and picture (B) of the novel environment test setup with the release tube attached on the left side and a coconut shelter in the open area.

We collected data on individual levels of boldness and exploration using a novel environment test (NET) (cf. Carter et al., 2013; Peignier et al. 2022). The setup (Figure 1) consisted of a cooler box (50 × 25 × 29 cm), with a 10 cm PVC tube attached on one side of the box (hereafter ‘release tube’). The floor of the box was visually divided into 40 squares (5 × 5 cm). An opaque sliding door separated the box from the release tube, so that it provided a safe, dark environment where the frog could calm down after being caught. In the lid of the box, we installed a wide-angle video camera (Hero Black 5, GoPro) and two elongated, battery powered LED lights (LUMIstixx, Osram/Ledvance, Garching, Germany). We also placed a coconut shelter in the box, similar to the one in the home terrarium, to hide part of the novel environment and motivate the frog to enter it. The position of the coconut shelter was the same for all individuals within each repetition, but varied between repetitions.

We ran experiments from 0900 to 1700. At the beginning of each trial, we caught a male in its home terrarium and placed it in the release tube for about 10 min to recover from capture. We then switched on the lights and camera, closed the lid of the box and opened the sliding door for 25 min. This allowed individuals to stay in the release tube or return to it at any moment after entering the box. As temperature varied during the day, we noted the exact ambient temperature (in °C) at the beginning of each trial. At the end of a trial, the individual was put back in its home terrarium and the setup was cleaned to remove potential chemical cues.

We analysed video recordings using the coding software BORIS (Friard et al., 2016). We coded (i) whether individuals entered the box (1) or not (0), (ii) the latency to leave the release tube (i.e., time until the individual’s entire body was inside the novel environment, in s), (iii) the time spent in the box (i.e., when the individual was in the open area of the box and not hiding under the coconut shelter, in s) and (iv) the number of visited squares in the box. Males who did not leave the release tube (*N* = 44/108 observations), were given a censored value of 1500 s for the latency to leave the release tube (i.e., total duration of the experiment), 0 s for the time spent in the box and 0 for the number of visited squares. A previous study already confirmed that both personality traits, boldness and exploration, can be distinctly measured by using this setup: ‘boldness’ affected the latency to leave a shelter and the probability to enter the novel environment, while ‘exploration’ affected the area visited in the novel environment (Peignier et al., 2022).

### 2.4 Habitat choice test

**Figure 2:**
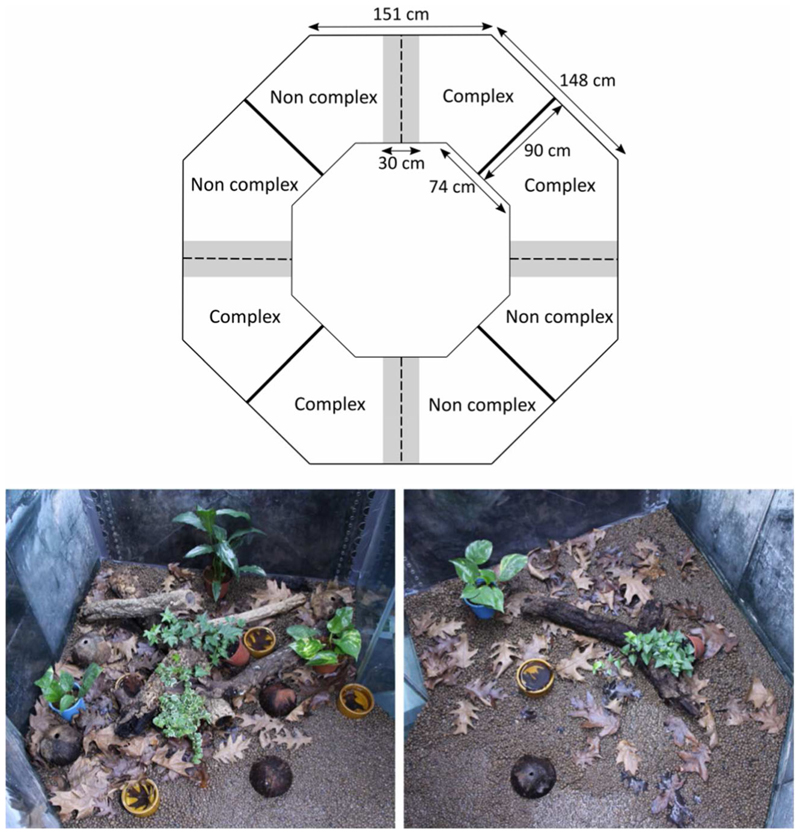
Scheme of the habitat choice test (top) and pictures of the complex (bottom left) and non complex (bottom right) habitats. The ring tank setup for the habitat choice test was split in four arenas separated by black walls (bold lines) to prevent any visual contact between males. Each arena was provided with a complex and a non-complex habitat of equal size that were visually separated for the analysis (dashed line). In the middle of each arena, an empty area (grey) served to release the frog at the beginning fo the test. In the complex habitat (bottom left), 80 to 90% of the clay pebbles were covered by a mix of leaves, plants, wood branches, water bowls and coconut shelters whereas in the non-complex habitat (bottom right), 10 to 20% of the clay pebbles were covered by a mix of the same components.

To investigate if males prefer different levels of habitat complexity according to their personality, we used a two-choice test presenting each male two habitats with variable complexity. Experiments took place in an 8000 litre ring tank. The soil of the ring tank was covered with clay pebbles and divided in four 2.4 m^2^ arenas allowing us to test four individuals at the same time (Figure 2). The arenas were separated by black walls preventing any visual contact between males. In each arena, the ‘complex’ and the ‘non-complex’ habitat area were separated by an empty area (30 cm wide, Figure 2). In the complex habitat, 80–90% of the clay pebbles were covered by a mix of oak leaves, plants, wood branches, water bowls and coconut shelters whereas in the non-complex habitat, only 10–20% of the clay pebbles were covered by a mix of the same components (Figure 2).

Before the start of the trials, the raining system was switched on for 10 minutes to ensure similar humidity levels across all trials. We caught males in their home terraria and placed them in a release tube in the middle area without the possibility to see nor access the habitats. We switched on a speaker broadcasting a recording of the ambient background noise in the housing room (i.e., consisting of advertisement calls of several males). We left the speaker playing for the entire duration of the trial to mimic a natural environment and stimulate the focal males to settle in a territory in the habitat choice setup. After a break of 15 minutes to recover from the stress of the capture, we lifted the release tube and switched on the video cameras to record the focal frogs’ behaviour during the next eight hours. At 1800, we caught the frogs and put them back in their home terraria. We switched on the raining system for five minutes to remove chemical cues from the setup. All experiments were conducted at a constant temperature of 29°C.

The first 30 minutes of the recordings were not considered in the analysis to allow individuals to acclimate to the new environment. We analysed only the subsequent 7.5 h of video recordings. For the analysis, the setup was split in two equal areas (‘complex’ versus ‘non-complex’ habitat), and we used the software BORIS (Friard et al., 2016) to code (i) the time spent in each habitat type (in s) and (ii) the number of times individuals crossed over from one habitat type to the other. The latter variable was used to identify if and how much individual males assess and compare the available habitat options.

### 2.5 Statistical analyses

We conducted all statistical analyses in R v.3.6.0 (R Core Team, 2020), using the integrated development environment RStudio v.1.2.1335 (RStudio Team, 2019). We used the function transformTukey to perform a constant transformation on variables which deviated from normality (i.e., calling duration during the activity trial, latency and time spent in the box during the NET).

First, we investigated whether origin (i.e., wild caught or captive bred), and body size influenced the behaviours measured in the activity trials (i.e., number of jumps and call duration) and in the NET (i.e., whether individuals entered the box, latency to leave the release tube, time spent in the box and number of visited squares). We additionally investigated the influence of temperature on the behaviours measured in the NET. For that, we fitted two generalized mixed effect models with a Poisson distribution and either the number of jumps or the number of visited squares in the NET as response variable. We also fitted a generalized mixed effect model with a binomial distribution, with whether individuals entered the box as response variable. Finally, we fitted three linear mixed effect models with either the call duration, the latency to leave the release tube or the time spent in the box (all transformed) as response variable. In these six models we added origin, and body size as fixed effects and male ID as random effect. We also added temperature as fixed effect in the four models with the behaviours measured in the NET as response variable.

To assess the amount of behavioural variation in the population due to inter-individual variation, we estimated the repeatability of each behaviour measured during the activity trial and the NET with the ‘rptR’ package (Stoffel et al., 2017). We considered behaviours to be repeatable if the 95% confidence interval (CI) did not overlap zero. We estimated repeatability from models fitted with a Gaussian error distribution for the call duration, the latency to leave the release tube and the time spent in the box (all transformed). We estimated repeatability from models fitted with a Poisson error distribution for the number of jumps and the number of visited squares in the NET, and from models fitted with a binary distribution for whether individuals entered the box. We included ID as a random effect in all models.

We also checked whether individuals chose one habitat over another based on its complexity. For that, we first checked data for normal distribution with a Shapiro-Wilk test (statistic = 0.49958, *p* < 0.001). We used a Wilcoxon one sample test comparing the time spent in the complex habitat to half of the total trial duration (13 500 s) to investigate if males made a choice (i.e., if time spent in a given habitat significantly deviates from what is expected by chance). Then, we investigated the influence of personality traits on males decision and movement in a territory settlement context. We built a generalized linear model and a linear model using the ‘lme4’ package (Bates et al., 2016). The generalized linear model followed a Poisson distribution and had the number of times individuals crossed over from one habitat type to the other as response variable. The linear model followed a Gaussian distribution and had the time spent in the complex habitat (transformed with the function transformTukey following a constant transformation) as response variable. For the fixed effects, we only used behaviours that were repeatable according to the repeatability estimations previously done (i.e., number of jumps as a proxy of activity, latency to leave the release tube as a proxy of boldness, and time spent in the box and mean number of visited squares in the box as proxies of exploration). We added the best linear unbiased predictors (scaled BLUPs, extracted from random intercept models) of these behaviours as fixed effects in the two models.

### 2.6 Ethics

The frogs used in this experiment belong to an ex-situ laboratory population at the animal care facility of the University of Bern. Original stock for this population, including all animals used for this study, was sampled in and exported from French Guiana in compliance with all legal requirements from the responsible French authorities (DIREN: Arrêté n°82 du 10.08.2012 and Arrêté n°4 du 14.01.2013). All testing was approved by the Suisse Federal Food Safety and Veterinary Office (National No. 33232, Cantonal No. BE144/2020). Captive conditions were approved by the Suisse Federal Food Safety and Veterinary Office (Laboratory animal husbandry license: No. BE4/11). We followed the guidelines laid out by the ASAB for the treatment of animals in behavioural research and Teaching (Asab, 2020) and the ARRIVE guidelines (Percie du Sert et al., 2020).

## 3 Results

### 3.1 Personality

There was no influence of origin and body size on any of the behaviours measured during the activity trials and the NET. There was also no influence of temperature on the behaviours measured in the NET (Table 1). The number of jumps measured during the activity trials was repeatable (Table 2), while the call duration measured during the activity trials was not (Table 2). Among the measures taken during the NET, all but the probably of individuals to enter the box turned out to be repeatable and ranged from 0.23 to 0.34 (Table 2).

### 3.2 Habitat choice test

**Figure 3:**
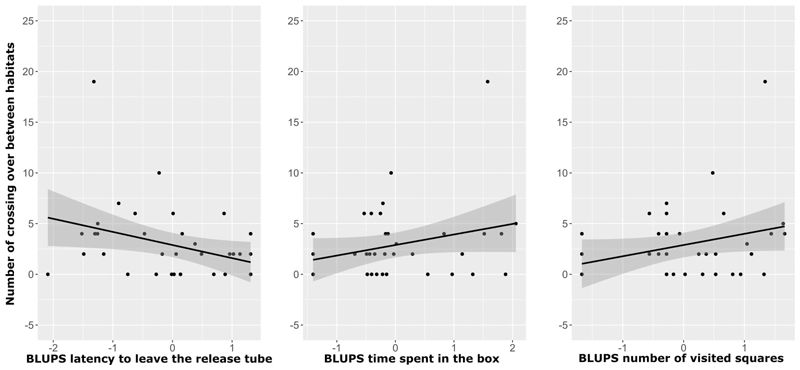
Plots presenting the link between behaviours measured in the novel environment test and the number of times an individual crossed from one habitat to the other in the habitat choice test. The BLUPs for the latency to leave the release tube (proxy for boldness, A), the time spent in the box (e.g., not hiding in the coconut shelter; proxy for exploration, B) and the number of squares visited (proxy for exploration, C) are presented. Each dot represents an individual, the black line represents the regression line and the grey surrounding area represents the 95% confidence interval.

During the habitat choice test, 23 out of 36 individuals explored both habitat types by crossing over from one habitat to the other. However, all individuals spent significantly more time in the complex habitat than in the non-complex one (Wilcoxon one sample test: *V* = 665, *p* < 0.001). The number of times individuals crossed over from one habitat type to the other was influenced by personality (Table 3). Individuals who were bolder (i.e. exited faster the release tube) and more explorative (i.e. stayed longer and visited more squares) in the NET, also crossed more often from one habitat type to the other in the choice test (Table 3; Figure 3).

## 4 Discussion

In the present study we investigated if habitat selection is driven by animal personality in the poison frog *Allobates femoralis*. By using a novel environment test and assessing individual activity patterns, we confirmed the existence of personality along the active/passive, bold/shy, and exploration/avoidance axes in our laboratory frog population. We show that the repeatability of the variables measured (ranging from 0.23 to 0.44) was in the lower range of what has been found in most personality studies in other taxa so far (mean = 0.37, 95%CI = 0.35,0.38) (Bell et al., 2009). However, our repeatability results are consistent with previous findings in amphibians (Brodin et al., 2013; Maes et al., 2013; Gifford et al., 2014; González-Bernal et al., 2014), and with what has been measured in wild *A. femoralis* (Peignier et al., 2022). The somewhat low repeatability in the present study is not surprising since ectotherms (compared to endotherms) and captive individuals (compared to wild populations) are typically less repeatable in their behaviours (Bell et al., 2009).

We also tested if habitat selection is driven by personality type using a two-choice test opposing two habitats with different level of complexity. When individuals enter a novel environment, they could either randomly disperse, all select the same environment (e.g., the environment that offers greatest resources) or select a habitat to match their personality (Holtmann et al., 2017). We expected shy individuals to select complex habitats (with more places to hide) and bold individuals to prefer non-complex habitats (where they are easier to spot for females). In our study, we observed that all individuals spent significantly more time in the complex compared to the non-complex habitat, regardless of their personality.

Territories with complex structures might in general represent an attractive habitat for male poison frogs. Indeed, vegetation composition plays a major role in the ecology of *A. femoralis*. During the reproductive season, males call from perches to both repel male competitors and attract females (Narins et al., 2003). Once the courtship initiated with the female, eggs are laid in the leaf litter (Ringler et al., 2012; Fischer et al., 2020). Therefore, choosing a complex habitat would be relevant regarding the species’ ecology, as it offers more resources such as more plants and branches to perch and hide, more leaf litter to hide and lay clutches. We cannot rule out that the simple habitat we offered was eventually too scarcely vegetated to be considered attractive, or that frogs evaluate a territory based on the combination of its natural and social characteristics (e.g., number of males or females around). However, our study design was chosen to specifically test for habitat preferences in the absence of any social cues, such as the presence of conspecifics. We speculate that in the wild, the distribution of certain personalities across the habitat is the result of a combination of different factors, including individual preferences and social interactions. If certain personalities have a competitive advantage when choosing a territory, this could also lead to a habitat type by personality correlation. Future studies should look into the relative contribution of social interactions and individual preferences on habitat choice and territory settlement.

Finally, we found that bolder and more explorative individuals were more likely to cross between habitats in the choice test. While this was not initially the main focus of our study, this result suggests that the behaviours measured in the NET indeed reflect exploration and boldness related behaviours measured in a more naturalistic setup. Animal personality is currently an omnipresent topic in animal behaviour reserach, and as such has been highly contested (cf. Beekman & Jordan, 2017). One of the main criticism is whether the use of artificial setups, such as a novel environment, really allows to measure behavioural patterns that reflect common behaviours of the species under natural conditions. Our results demonstrate that exploration behaviour measured in a highly artificial setup indeed reflect exploration behaviour in a more naturalistic setup. We encourage more studies to investigate personality traits across different experimental complexities and settings to assure the ecological relevance of the behaviours measured.

## Figures and Tables

**Table 1:** Results of the (generalized) linear mixed effect models investigating the influence of body size and origin on behaviours measured during the activity trial and of body size, origin and temperature on behaviours measured during the NET.

	Number of jumps		Call duration		Probably to enter the box		Latency to leave the release tube		Time spent in the box		Number of visited squares	
Fixed effects (Estimate ± SE | *p *value)												
(Intercept)	−3.02±6.94	0.664	10.59±10.99	0.335	−4.58±8.03	0.569	13.1±18.17	0.471	−3.60±5.35	0.501	−5.10±5.34	0.339
Body size	1.51±2.41	0.532	−2.65±3.82	0.488	1.41±2.23	0.528	3.63±5.21	0.487	1.18±1.54	0.444	2.05±1.81	0.258
Origin	0.73±0.64	0.260	0.99±1.02	0.331	−0.78±0.62	0.205	0.80±1.40	0.570	−0.50±0.41	0.229	−0.22±0.49	0.647
Temperature					0.06±0.19	0.763	−0.49±0.40	0.212	0.08±0.12	0.489	0.01±0.04	0.718
Random effects (Estimate ± SD)												
ID	3.12±1.77		3.14±1.77		1.05±1.02		8.11±2.85		0.74±0.86		1.69±1.3	
Residual			15.59±3.95				22.27±4.72		1.85±1.36			

*N* = 108 observations on 36 individuals.

**Table 2:** Repeatability (*R*) and confidence intervals (CI) of the behaviours measured during the activity trials and the NET.

Test	Variable	*R*	95% CI	*p*
Activity trials				
	Number of jumps	0.44	[0.01, 0.17]	<0.001*
	Call duration ^a^	0.16	[0, 0.37]	0.064
NET				
	Probability to go in the NET	0.22	[0, 0.45]	0.022
	Latency to leave the release tube ^a^	0.23	[0.007, 0.43]	0.01^*^
	Time spent inside the box ^a^	0.29	[0.06, 0.48]	0.004^*^
	Number of visited squares	0.34	[0.02, 0.56]	0.004^*^

Significant *p* values (<0.05) are indicated with asterisks.

a Variables which deviated from normality and were transformed with a constant transformation. *N* = 36 individuals.

**Table 3:** Results of the (generalized) linear models investigating the link between personality traits and habitat choice.

Response variable	Fixed effects	Estimate	SE	*p*
Number of crossing over from one habitat to the other				
	(Intercept)	−0.89	0.12	<0.001*
	BLUPs number of jumps	−0.05	0.12	0.651
	BLUPs latency to leave the release tube	−0.59	0.17	<0.001^*^
	BLUPs time spent in the box	−0.50	0.25	0.041^*^
	BLUPs number of visited squares	0.62	0.21	0.003^*^
Time spent in the complex habitat			
	(Intercept)	1.17 · 10^44^	8.08 · 10^42^	<0.001^*^
	BLUPs number of jumps	1.49 · 10^43^	8.58 · 10^42^	0.093
	BLUPs latency to leave the release tube	9.46 · 10^42^	1.36 · 10^43^	0.492
	BLUPs time spent in the box	3.73 · 10^42^	2.07 · 10^43^	0.858
	BLUPs number of visited squares	−7.58 · 10^42^	1.56 · 10^43^	0.631

Model estimates, standard error, and *p* values are presented. Significant *p*-values (<0.05) are indicated with asterisks. *N* = 36 individuals.
